# Tregenna Biddulph Goss

**Published:** 1910-12

**Authors:** 


### Obituary Notice.

### TREGENNA BIDDULPH GOSS, M.R.C.S., L.S.A.

It is with very great regret that we have to record the death of Mr. T. B. Goss at the age of 73, which occurred at Bath on November 20th, where he had been in practice for forty-two years. Mr. Goss was known to many members of the profession in consequence of the great regularity with which he attended medical meetings, both in Bath and Bristol, and to know him was to like him. His popularity with all in Bath was very great, and he was never known to have a dispute with any member of the profession. His kindly and genial disposition and his deference to the opinion of others, whilst maintaining his own views, enabled him to live a prosperous professional life in perfect friendship with all his brethren. He was elected President of the Bath and Bristol Branch of the British Medical Association in 1892.

His sympathy with his patients, and his kindness to those of them who had no means of repaying him, are gratefully remembered by many who now mourn the loss of a doctor who never spared his time when his help was called for, and a warm personal friend who could always be relied upon when his advice was needed.

